# Establishing the role of the FES tyrosine kinase in the pathogenesis, pathophysiology, and severity of sepsis and its outcomes

**DOI:** 10.3389/fimmu.2023.1145826

**Published:** 2023-04-14

**Authors:** Brian J. Laight, Natasha A. Jawa, Kathrin Tyryshkin, David M. Maslove, J. Gordon Boyd, Peter A. Greer

**Affiliations:** ^1^ Department of Pathology and Molecular Medicine, Faculty of Health Sciences, Queen’s University, Kingston, Ontario, ON, Canada; ^2^ School of Medicine, Faculty of Health Sciences, Queen’s University, Kingston, Ontario, ON, Canada; ^3^ Queen’s Cancer Research Institute, Queen’s University, Kingston, Ontario, ON, Canada; ^4^ Centre for Neuroscience Studies, Faculty of Health Sciences, Queen’s University, Kingston, Ontario, ON, Canada; ^5^ School of Computing, Queen’s University, Kingston, Ontario, ON, Canada; ^6^ Division of Medicine and Critical Care Medicine, Department of Medicine, Faculty of Health Sciences, Queen’s University, Kingston, Ontario, ON, Canada; ^7^ Departments of Medicine and Critical Care Medicine, Kingston General Hospital, Kingston, Ontario, ON, Canada

**Keywords:** sepsis, innate immunity, dysregulated inflammation, coagulation, personalized medicine

## Abstract

**Introduction:**

Sepsis is a result of initial over-activation of the immune system in response to an infection or trauma that results in reduced blood flow and life-threatening end-organ damage, followed by suppression of the immune system that prevents proper clearance of the infection or trauma. Because of this, therapies that not only limit the activation of the immune system early on, but also improve blood flow to crucial organs and reactivate the immune system in late-stage sepsis, may be effective treatments. The tyrosine kinase FES may fulfill this role. FES is present in immune cells and serves to limit immune system activation. We hypothesize that by enhancing FES in early sepsis and inhibiting its effects in late sepsis, the severity and outcome of septic illness can be improved.

**Methods and analysis:**

*In vitro* and *in vivo* modeling will be performed to determine the degree of inflammatory signaling, cytokine production, and neutrophil extracellular trap (NET) formation that occurs in wild-type (WT) and FES knockout (*FES^-/-^*) mice. Clinically available treatments known to enhance or inhibit FES expression (lorlatinib and decitabine, respectively), will be used to explore the impact of early *vs.* late FES modulation on outcomes in WT mice. Bioinformatic analysis will be performed to examine *FES* expression levels in RNA transcriptomic data from sepsis patient cohorts, and correlate *FES* expression data with clinical outcomes (diagnosis of sepsis, illness severity, hospital length-of-stay).

**Ethics and dissemination:**

Ethics approval pending from the Queen’s University Health Sciences & Affiliated Teaching Hospitals Research Ethics Board. Results will be disseminated through scientific publications and through lay summaries to patients and families.

## Highlights

•This will be the first study to explore the utility of FES modulation in the treatment of sepsis.
•This study will illustrate the importance of differential regulation of sepsis at both early and late timepoints.
•This study will employ *in vivo, in vitro*, and clinical research methodologies to elucidate the role of FES in sepsis.
•There exists the potential for off-target effects of the drugs used in this study that may complicate the interpretation of results or outcomes, as they are not specific enhancers or inhibitors of FES.

## Introduction

Sepsis is the leading cause of death among hospitalized patients in North America, and is present in over 50% of adult hospitalizations resulting in death or discharge to hospice care (1). Globally, sepsis accounts for 19.7% of all deaths (2), and is the most expensive healthcare problem requiring in-hospital treatment (3), costing the healthcare system approximately $1 billion per year in Ontario alone ($670 million for severe cases, $420 million for non-severe cases) (4). Importantly, the consequences of sepsis extend beyond the duration of the patient’s hospitalization, with lasting impairments being increasingly recognized as comprising “post-sepsis syndrome” (5); resulting in additional costs to the healthcare system for auxiliary care after discharge. Targeted treatments for sepsis are therefore urgently needed to simultaneously improve patient outcomes and alleviate the burden of sepsis on global healthcare systems.

Sepsis is characterized by widespread inflammation and life-threatening organ dysfunction, resulting from dysregulation of the patient’s response to infection and coagulation cascades (6, 7). Specifically, sepsis occurs upon disruption of the tightly regulated balance of pro- and anti-inflammatory mediators activated in response to pathogen- (e.g., virus, bacteria) or damage-associated molecular patterns (e.g., trauma; PAMPs and DAMPs, respectively) (8). Binding of PAMPs and DAMPs to toll-like receptors (TLRs) on the surface of antigen presenting cells (APCs) and monocytes causes nuclear translocation of nuclear factor kappa light chain enhancer of activated B cells (NF-κB), resulting in expression of genes encoding pro-inflammatory cytokines, tumor necrosis factor (TNF)-α, interferons, and components of complement and coagulation pathways (2). Clinically, activation of these pathways results in hypercoagulation, endothelial dysfunction, and ultimately to end organ damage (2).

While sepsis pathophysiology remains to be fully elucidated, we have previously shown that the FES kinase, which is highly expressed in APCs, plays a role in limiting over-stimulation of the innate immune system (9). *FES* knockout mice are hypersensitive to endotoxin lipopolysaccharide (LPS), a TLR-4 agonist, as a result of overactive TLR-4 signaling-induced innate immune responses (10) including increased transcription of genes encoding pro-inflammatory mediators, such as TNF-α (9). Our group and others have implicated FES as a regulator of TLR-4 signaling (9, 11). TLR-4 is also a critical inducer of both platelet-dependent and -independent NET formation, where neutrophils and platelets express both FES and TLR-4. Hyperactive TLR-4 signaling may therefore exacerbate the immune response and coagulation in severe sepsis (12, 13). Furthermore, *FES* knockout (*FES^-/-^*) mice demonstrate dysregulation of signal transducer and activator of transcription 3 (STAT3) (13), which plays a vital role in the pathophysiology of sepsis by initiating endothelial dysfunction, vasoplegia, coagulopathy, and multi-organ failure (14, 15). FES may also contribute to thrombin formation (16), which is known to contribute to hypercoagulation during the early inflammatory stage of sepsis (17).

There are currently no approved targeted molecular therapies available for the treatment of sepsis (6). The mainstay of treatment for sepsis involves management of infection, optimizing the patient’s fluid balance, improving hemodynamic stability, and sedative management; however, there is controversy over whether these interventions contribute to reduced mortality and morbidity in this cohort (3, 6, 18). Furthermore, early pre-clinical trials employing anti-TNF antibodies or corticosteroids to block proinflammatory cytokine cascades have since been shown to be ineffective in larger and later-stage clinical trials (3, 6). Identifying and targeting the key events and their regulators that precede these dysregulated immune responses characteristic of sepsis is therefore critical to optimize patients’ responses to systemic infection (6).

Due to its expression in innate immune cells and its involvement in regulating TLR-4 signaling (9, 11), we expect that increased FES expression will limit hyper-inflammatory phenotypes of immune cells (9, 11), reduce platelet aggregation (19), and we hypothesize it will also reduce NET formation (12, 20, 21). Consequently, targeting FES may allow for specific inhibition of two key aspects underlying the pathophysiology of sepsis: the coagulation cascade and the innate immune system. This study aims to elucidate the role of FES in sepsis pathophysiology using both an established mouse model of sepsis and a repository of data from patients with sepsis, as a first step toward being able to develop targeted immune-based therapies to enhance patient care and improve outcomes.

## Overarching hypothesis

**Figure 1:**
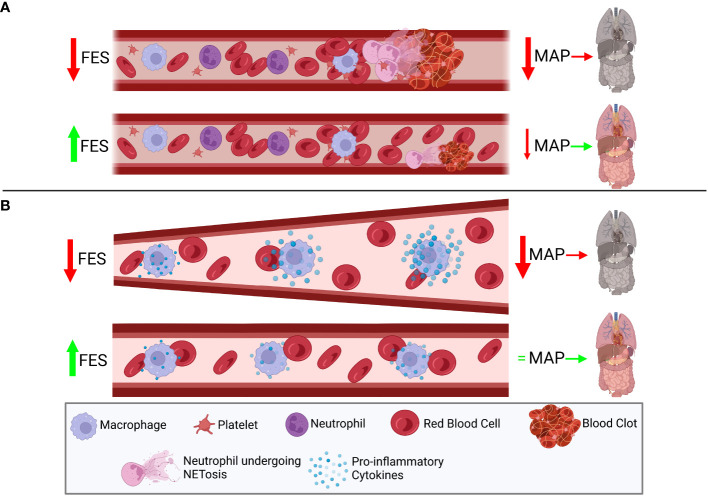
Decreased FES levels may correlate with worse septic outcomes by modulating host immune and coagulation pathways. **(A)** FES has been shown to play a role in limiting coagulation cascades through modulation of platelets and we hypothesize a role for FES in decreasing NETosis from neutrophils. Therefore, low FES expression is hypothesized to correlate with reduced lysis of blood clots, and an increase in NETosis in both a platelet-dependent and –independent manner, thus leading to reduced mean arterial pressure (MAP), leading to end organ damage. However, in patients with high FES expression, there is hypothesized to be reduced NETosis, and increased disaggregation of platelets, leading to a less dramatic impact on MAP, and improved perfusion of end organs. **(B)** FES has been shown to suppress the toll-like receptor (TLR) 4 signaling cascade, which leads to the production of pro-inflammatory cytokines regulated by NF-κB and IRF3. In patients with low FES expression, it is hypothesized that this signaling cascade will be poorly regulated, leading to the over-activation of the TLR4 cascade, stimulated by components of bacterial cell walls (lipopolysaccharide) or in response to tissue damage (high mobility group box 1), and ultimately leading to the over-production of inflammatory cytokines. This production of excess cytokines can lead to vasodilation, leading to reduced MAP, and reducing perfusion to end organs. However, with high FES expression, this cascade is tightly regulated, and the over-production of pro-inflammatory cytokines is limited, reducing changes in vasodilation, and preventing drastic changes in MAP, thus protecting end organs. Created with BioRender.com.

**Figure 2:**
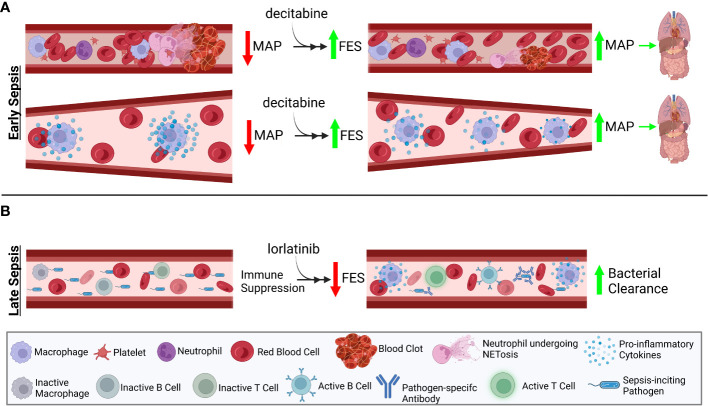
Therapeutic efficacy of FES expression level changes is stage-dependent. Sepsis can be separated into two stages: early stage, characterized by overactive immune responses leading to decreased end organ perfusion; and late stage, characterized by immune suppression, preventing the efficient clearance of the sepsis-inciting event. **(A)** Due to the over-stimulation of the immune system in early sepsis, treatment with decitabine, known to induce increased *FES* expression, will prevent overactivation of the immune system. **(B)** In contrast, overcoming the characteristic late-stage immunosuppression by treatment with lorlatinib, an inhibitor of FES activity, will promote greater pro-inflammatory cytokine production from immune cells. This increase in immune activation will aid in resolving the inciting event of sepsis. Created with BioRender.com.

We hypothesize that FES expression is related to outcomes in *in vivo* and *in vitro* models of sepsis, as well as in clinical populations of patients with sepsis. We further hypothesize that modulating FES can be used as a method of improving sepsis outcomes. **Figure 1** illustrates the hypothesized role of FES in the pathophysiology of sepsis. **Figure 2** illustrates how FES regulation could be used therapeutically to treat sepsis.

## Aims and specific hypotheses

### Cell types responsible for sepsis severity and their effect on sepsis outcomes

This study aims to determine the cell types responsible for increased sepsis severity and their effects on outcomes in WT or *FES^-/-^* mice. We hypothesize that FES limits over-activation of innate immune cells and suppresses vascular hypercoagulation. We expect that *FES^-/-^* immune cells will take on a hyper-inflammatory phenotype when stimulated with infectious or damaging agents and will produce increased levels of pro-inflammatory cytokines (such as Interleukin (IL) -1, IL-6, Tumor Necrosis Factor (TNF)-α) and NETs relative to WT mice. *In vivo*, we expect to find an increase in both immune cell numbers and their degree of activation, as well as increased coagulation and end-organ damage in septic *FES^-/-^* mice compared to WT.

### Timing of FES regulation on sepsis outcomes

This study will aim to evaluate whether FES regulation, and the timing of FES regulation, can affect sepsis outcomes. Early sepsis pathogenesis is characterized by hyperinflammation and coagulation, while late sepsis pathogenesis is characterized by immune suppression and organ dysfunction. It is hypothesized that the observed late-stage immunosuppression is as a commensal response to the initial overactive immune response. The early hyper-inflammatory stage of sepsis is characterized by increased immune cell activation and production proinflammatory cytokine secretion such as IL-1, IL-6, and TNF-α (22), a dangerous condition known as cytokine storm (23). This late stage is characterized by production of anti-inflammatory cytokines, such as IL-10 and Transforming Growth Factor-β (TGF-β), which serve to repress the overstimulated immune cells, reduced antigen presenting cells functions, lymphocyte anergy and apoptosis, and reduced responsiveness to activating stimuli, such as LPS (24). Simultaneous inflammation and immunosuppression is now being described in late-stage sepsis (22), suggesting that while there may be immunostimulatory molecules circulating, immune cells may be unable to appropriately respond to these stimuli. This provides additional rational for regulating FES, as it plays a role in limiting innate immune cell activation in response to stimulatory molecules such as LPS. We therefore hypothesize that artificially enhancing FES activation will improve early-stage sepsis, and that inhibiting FES will improve outcomes in late-stage sepsis. **Figure 2** illustrates the timeline of sepsis and the expected impact of FES regulation at each stage of illness.

### Association between FES expression and clinical sepsis presentation and outcomes

This study will seek to determine whether the RNA expression levels of FES are associated with the incidence, severity, and outcome of sepsis using bioinformatic analysis. We expect that FES will be upregulated in early-stage and downregulated in late-stage sepsis. Furthermore, we hypothesize that decreased *FES* RNA levels will be associated with receiving a clinical diagnosis of sepsis, increased illness severity, and poorer clinical outcomes including greater risk for ICU mortality and increased length of hospitalization.

Furthermore, we aim to determine whether clinical sepsis outcomes are correlated with single nucleotide polymorphisms (SNPs) associated with differential *FES* expression. Recently, two SNPs have been associated with reduced *FES* expression: rs17514846, located within in the 5’ proximal region of the neighbouring gene *FURIN*, and rs1894401, located within the *FES* gene. The presence or absence of these two SNPs will be correlated with clinical outcomes in a cohort of sepsis patients (25).

## Methods and analysis

### Patient and public involvement

Patient partners who have recovered from sepsis will be recruited from the ICU Follow-Up Clinic at Kingston Health Sciences Centre (Kingston, Ontario). Patient partners will help to guide decision-making around the clinical outcomes that are most important to study, such that our analysis can be focused on patient-centred outcomes.

### Study design and setting

To expand our understanding of FES involvement in mediating sepsis, we will perform *in vitro* and *in vivo* modeling to elucidate the degree of inflammatory signaling, cytokine production, and NET formation that occurs in wild-type (WT) and *FES* knockout (*FES^-/-^*) mice. Using clinically available treatments that either enhance *FES* expression or inhibit FES activity, we will explore the utility of early *vs.* late FES modulation on outcomes in WT mice. Finally, we will explore the clinical relevance of FES in the pathogenesis of sepsis by using bioinformatic analyses to examine *FES* expression levels in RNA transcriptomic data from publicly available cohorts of patients with sepsis and healthy controls and correlating this with clinical outcomes. All analyses will take place in the Queen’s Cancer Research Institute facility at Queen’s University (for experimental laboratory-based aims), or the Centre for Advanced Computing (for the clinical bioinformatics aims).

### *In vitro* methodology

**Figure 3:**
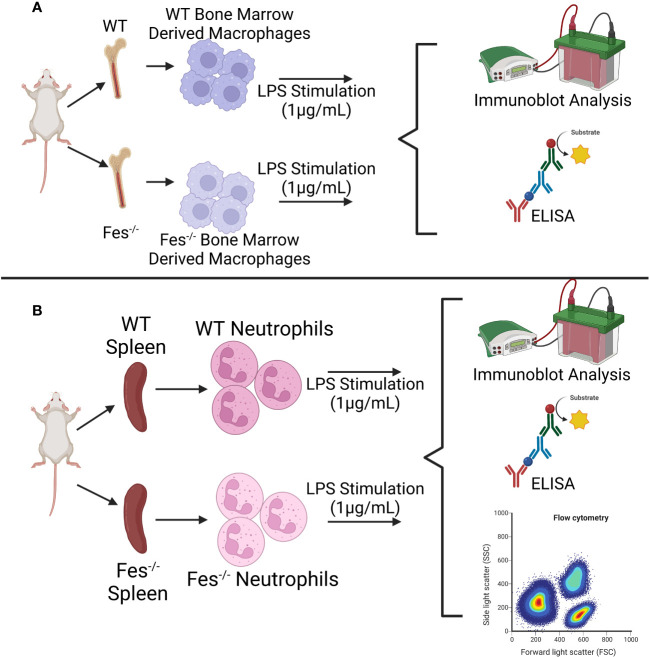
Graphical depiction of *in vitro* methodology. **(A)** Wildtype (WT) or FES^-/-^ bone marrow derived macrophages will be stimulated with LPS or other stimulating agents and analyzed for differential signaling cascade activation (immunoblot analysis) and cytokine production (enzyme-linked immunosorbent assay (ELISA)). **(B)** WT or FES^-/-^ neutrophils isolated from the spleen will be stimulated with LPS or other stimulating agents and analyzed for differential signaling cascade activation (immunoblot analysis), cytokine production (ELISA), and neutrophil extracellular trap formation (flow cytometry). The same methodology will be followed with the addition of decitabine and lorlatinib. Created with BioRender.com.

Bone marrow derived macrophages (BMDMs) (26) or neutrophils (27) isolated from WT or *FES^-/-^* mice will be stimulated with infectious surrogates (LPS or PolyI:C), or a surrogate for tissue damage-mediated sepsis (High Mobility Group Box 1; HMGB1). The degree of inflammatory signaling (e.g., NF-κB and IRF-3 phosphorylation) will be assessed via immunoblot analysis. Supernatants will be analyzed by cytokine multiplex (28) methods to determine differential cytokine production in WT or *FES^-/-^* immune cells, and the type of cytokines (e.g., proinflammatory, such as IL-1, IL-6, IL-12, TNF-α, and Interferon-α/β (23); or anti-inflammatory, such as, IL-10 and TGF-β (29)) (**Figure 3A**). Finally, WT and *FES^-/-^* neutrophils will be evaluated for their ability to produce NETs using flow cytometry (30) (**Figure 3B**).

BMDMs and neutrophils isolated from WT or *FES^-/-^* mice will be pre-treated with either a drug that inhibits FES activity (*lorlatinib* (31)), or one that increases *FES* transcription (*decitabine* (32)). These cells will then be stimulated with LPS, PolyI:C, or HMGB1 to simulate the immune cell activation associated with sepsis, and the same methodology as in Aim 1 will be used to assess increased (in the case of lorlatinib treatment) or decreased (in the case of decitabine treatment) hyper-inflammatory signaling, cytokine production, and NET production.

### *In vivo* methodology

**Figure 4:**
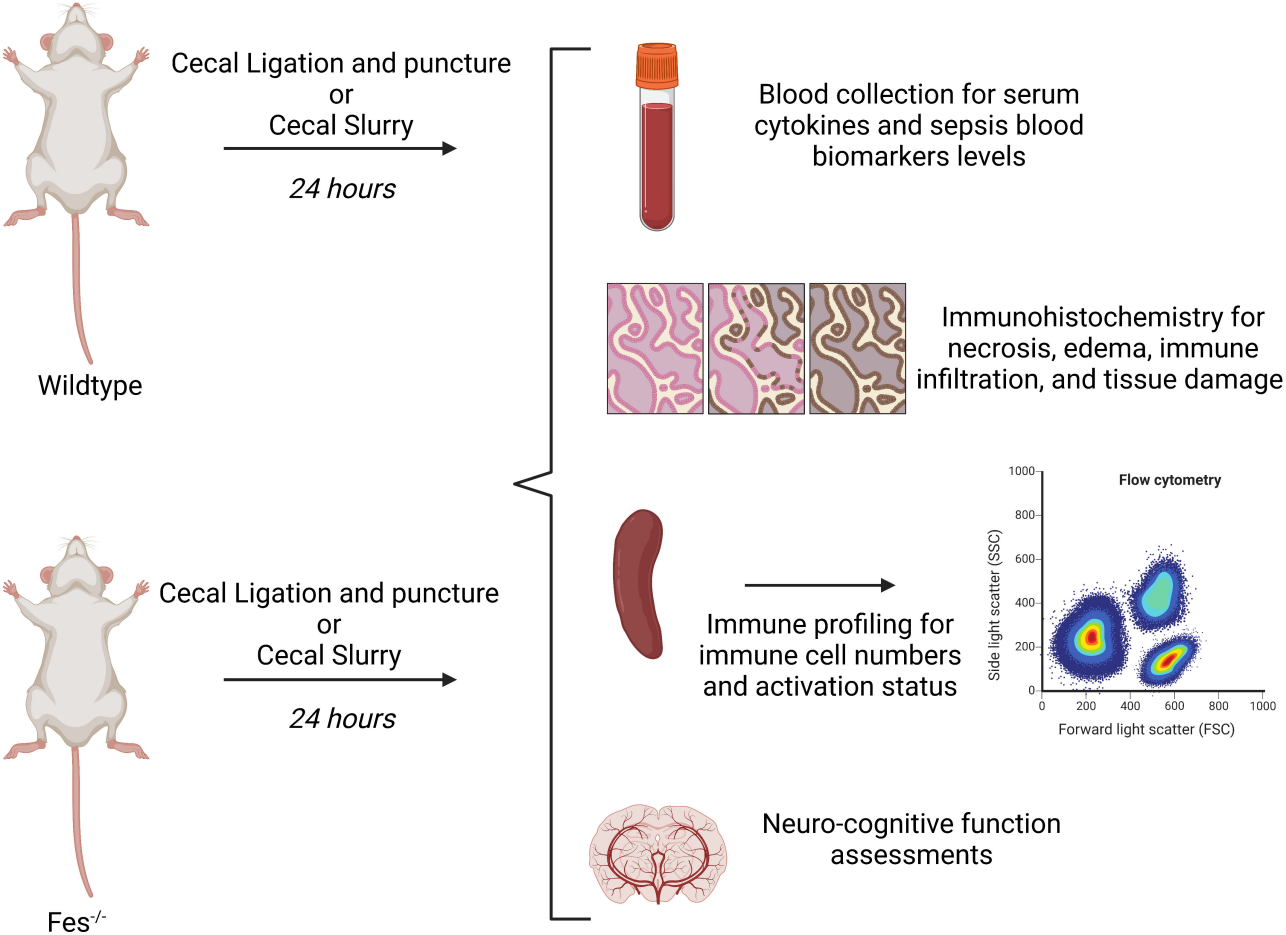
Graphical depiction of *in vivo* methodology. Wildtype (WT) or FES^-/-^ mice will be undergo a cecal ligation and puncture (CLP) or receive a cecal slurry injected intraperitoneally. 24 hours later, mice will be assessed for the following: neurologic function assessment, serum cytokine and biomarker analysis, immunohistochemistry, and spleen immune profiling. The same methodology will be used with the addition of decitabine or lorlatinib. Created with BioRender.com.

WT or *FES^-/-^* mice will be treated in one of two ways, either simulating different insults resulting in sepsis: 1) cecal ligation and puncture (CLP), the gold-standard sepsis model (33) or 2) a cecal slurry (CS) of 0.5mL of a solution at a fecal concentration of 45mg/mL (33–35). Following induction of sepsis, mice will be closely monitored and scored on the murine sepsis score (MSS), which assesses sepsis severity based on appearance, level of consciousness, activity, response to stimulus, eyes, respiration rate, and respiration quality (35). The MSS has been shown to have a specificity of 57%, and a sensitivity of 100% for predicting onset of severe sepsis and death following CS. Blood will be collected by cardiac puncture at the 24h time point and analyzed by cytokine multiplex to assess differential cytokine production, which will demonstrate differences in cytokine production from either WT or *FES^-/-^* genotypes and can also be correlated with sepsis severity (35). Additionally, the following biomarkers will be assayed from the blood, as these have been associated with sepsis in murine models: C-reactive protein (CRP), soluble triggering receptor expressed on myeloid cells-1 (sTERM-1), CD163, procalcitonin (PCT) and hypoxia-inducible factor (HIF-1α) by ELISA, where CD163 and procalcitonin can stratify mice further into a state of severe sepsis (36) (**Figure 4**). Spleens will be isolated, dissociated and analyzed by flow cytometry for the presence and activation of immune cells (i.e., macrophages, neutrophils, T cells, B cells), as well as NET formation (30). Finally, differences in clotting ability and end-organ damage between WT and *FES^-/-^* mice will be assessed by histopathological analysis of hematoxylin & eosin-stained necropsy tissues. Briefly, livers, kidneys, small intestines, lungs, and brains will be assessed for necrosis, edema, accumulation of immune cells within tissues (e.g. macrophages, neutrophils, T-cells) and signs of damage to the tissues (e.g., loss of goblet cells and villi in small intestines, debris, and loss of organization and structure of epithelium) (35). As CS with a fecal concentration of 45mg/mL is associated with a 40% survival in C57BL/6 wildtype mice, mice will be assessed for neurocognitive impairments: a significant sequalae of post-sepsis syndrome (37, 38) A recent systematic review listed several common forms of cognitive impairment found in pre-clinical models of sepsis (e.g., aversive memory, learning, locomotor and exploratory activities, short-term and long-term memories), which may mimic what happens in patient populations (37). Therefore, any surviving mice will be subjected to the Y-maze novel arm preference test (a measure of testing spatial memory and attention), buried food test (a measure of attention and organized thinking), attentional set-shifting task (a measure of attention and cognitive flexibility), and finally the open field test (a measure of motor performance) (39) (**Figure 4**). Following neurocognitive assessments, mice will be euthanized and assessed for hyper-inflammation, coagulation, and histopathology as described above.

To determine the effect of the timing of FES regulation on outcomes, WT or *FES^-/-^* mice will be subjected to either CLP or CS and treated with lorlatinib or decitabine at either 1h (mimicking regulation in early-stage sepsis) or 12h (mimicking regulation in late-stage sepsis). Hyper-inflammation, coagulation, histopathology, and neurocognition will be assessed at 24h, as previously mentioned. Finally, to determine differential susceptibility of WT mice to sepsis, we will perform an LD_50_ analysis of both early- and late-stage treatment regimens with either drug.

### Clinical and bioinformatics methodology

We have previously extracted RNA from whole blood samples in critically ill patients at various time points during their admission, as well as from healthy controls, as part of the “Prevention of nosocomial infections in critically ill patients with lactoferrin (PREVAIL)” study (NCT01996579). Briefly, blood was collected into PAXgene tubes, frozen, RNA extracted, and gene expression was profiled using the Affymetrix PrimeView microarray (40). Data on 69 patients and 21 healthy controls from this analysis is publicly available (41) and will be analyzed for the current study. Machine-learning approaches will be used to identify key variables associated with FES RNA expression levels, including a) *clinical diagnosis of sepsis*; b) *illness severity*, as measured by Acute Physiology and Chronic Health Evaluation II (APACHE II) and Sequential Organ Failure Assessment (SOFA) scores; and c) clinical outcomes including *hospital length of stay and ICU mortality*.

A publicly available dataset comprised of clinical outcome data as well as SNP analysis from genome wide association studies (GWAS) will be sought through collaborations with local and international experts in the field of sepsis genetics. Multivariable analysis will be used to determine the association between the two SNPs (rs17514846 and rs1894401) related to differential FES expression, and clinical, patient-centred outcomes. Additionally, we will apply for access to the United Kingdom BioBank (https://www.ukbiobank.ac.uk/), a large-scale database with in-depth genetic and clinical outcomes for a wide variety of diseases (including sepsis) in over half a million patients. This database currently has SNP and transcriptomic data available to be paired with clinical outcomes and will soon be releasing proteomic data which can be analyzed further to pair the two SNPs (rs17514846 and rs1894401) related to differential FES expression with protein expression, and clinical outcomes.

## Expected impact

These results will determine if FES modulates sepsis severity by preventing hyper-inflammation and suppressing coagulation cascades, providing a rationale for using FES regulation as a therapeutic strategy to reduce sepsis severity. The results of these experiments will further improve our understanding of the process by which NETs are produced, which has implications not only for sepsis but also for many other diseases.

In lorlatinib treated WT immune cells, we expect to see a phenocopy of the *FES^-/-^* immune cells, and prevention of inflammatory signaling with decitabine. Irrespective of the timing of treatment, we do not expect either lorlatinib or decitabine to affect sepsis severity in *FES^-/-^* mice (as there is no FES present to be regulated by either of these drugs). By contrast, in WT mice we expect to see increased septic severity following early lorlatinib treatment, and decreased septic severity following early decitabine treatment. We further expect to see decreased septic severity following late lorlatinib treatment (protective effect) and increased septic severity with late decitabine treatment (detrimental effect). These findings would support the notion that FES is a critical regulator of sepsis; and would further demonstrate that sepsis severity can be attenuated using currently available treatments by differentially modulating FES depending on the stage of a patient’s illness progression.

While it is our expectation that high FES expression levels in early sepsis and low FES expression levels in late sepsis will result in improved murine and patient outcomes, it is possible that this will not be the case. However, irrespective of the direction of the association, we will capitalize on the FES expression results found in order to improve *in vitro*, *in vivo* outcomes. For example, if we find that low FES expression is associated with improved outcomes in early sepsis (contrary to our hypothesis), we will expand upon this finding by seeking to reduce FES expression in our models of early sepsis. Similarly, if–contrary to our expectations–we find that high FES expression is associated with improved outcomes in late sepsis, we will aim to increase FES expression in our models of late sepsis and evaluate changes in outcomes seen as a result of this shift in expression. Additionally, if FES expression plays no role in sepsis severity, it will allow us to rule out a potential regulator heavily involved in many aspects of sepsis, which will help to narrow in on true regulators of sepsis severity and outcomes. We expect that *FES* expression levels on microarray analysis can be used alone or in combination with other genetic features as a biomarker to determine the presence, stage, severity, and outcome of sepsis. The heterogeneity of sepsis presentation and progression has long posed a problem for determining which patients will benefit from specific therapies, and the appropriate timing of these interventions. If we find that FES expression differs between time points in the progression of septic illness (i.e., between early- *vs*. late-stage sepsis), these results may then be used in future studies to help with targeting treatments toward specific subsets of patients who may differentially benefit from therapeutic interventions applied at different time points.

Finally, we expect that two SNPs associated with differential FES expression will be useful as prognostic biomarkers to stratify patients with sepsis based on risk for adverse outcomes that are relevant to patients, with the potential predictive value to inform FES-targeted treatments based on risk.

An important note about the involvement of FES in the pathophysiology is the association of SNP rs4957796, located within an intron of the FER gene (Fps/Fes related tyrosine kinase), with reduced mortality from sepsis (42), and reduced incidence of bloodstream infections, but increased mortality in those who do develop a bloodstream infection (43). FES and FER share similar biological functions such as platelet aggregation (19), mast cell activation (13, 44) and signaling of the immunosuppressive cytokine IL-10 (13), an important signalling pathway known to limit inflammation, whose function may have differing important roles during early- (important to limit the overactivation of the immune system) and late-stage (contribution to characteristic late-stage immunosuppression) sepsis. These observations may suggest a partial explanation to the improved outcomes in patients containing the SNP, rs4957796 (42) as well as the observations surrounding bloodstream infections (43). This hints that the impact of FER and potentially FES is double-edged sword where it is crucial to have the right amount of expression at the right time to have protective effects and not exacerbate the illness. If true, this would suggest a problem that the application of precision medicine might be able to overcome.

Sepsis is a disease that is constantly under investigation to find an effective treatment because of the major consequences on human life and healthcare burden. Unfortunately, there has not been much success towards this aim; over 100 randomized clinical trials which identify a target, and directly modulate that target, have failed (45), suggesting a need for novel approaches to treating sepsis. Not only do we believe we have identified a novel target capable of regulating multiple aspects of sepsis pathophysiology, but also the timing of intervention. This lends itself to identifying FES as a treatable trait (46), where, depending on the timing of intervention and the levels of FES expression or activity, may result in a different approach to treating a patient’s sepsis (upregulating FES expression or inhibiting FES activity).

## Significance

Sepsis is one of the greatest healthcare problems worldwide, causing significant mortality and costing the Canadian healthcare system billions of dollars annually (4). Even among those who survive, sepsis is associated with long-term morbidities and reduced quality of life (4, 47). This project will provide crucial insight into the pathophysiology underlying sepsis, laying the foundation for future work to manage the dangerous side effects of immunotherapy-based treatments, and study the effect of systemic FES inhibition on this vulnerable cohort.

## Author contributions

BL, DM, and PG designed the study. BL and NJ wrote the study protocol. BL, NJ, KT, DM, JB, and PG reviewed and revised the manuscript. All authors contributed to the article and approved the submitted version.

## References

[1] Rhee C Jones TM Hamad Y Pande A Varon J O’Brien C . Prevalence, underlying causes, and preventability of sepsis-associated mortality in us acute care hospitals. JAMA Network Open (2019) 2(2):e187571. doi: 10.1001/jamanetworkopen.2018.7571 30768188 PMC6484603

[2] Jarczak D Kluge S Nierhaus A . Sepsis-pathophysiology and therapeutic concepts. Front Med (Lausanne) (2021) 8:628302. doi: 10.3389/fmed.2021.628302 34055825 PMC8160230

[3] Gyawali B Ramakrishna K Dhamoon AS . Sepsis: The evolution in definition, pathophysiology, and management. SAGE Open Med (2019) 7:1–13. doi: 10.1177/2050312119835043 PMC6429642 30915218

[4] Farrah K McIntyre L Doig CJ Talarico R Taljaard M Krahn M . Sepsis-associated mortality, resource use, and healthcare costs: A propensity-matched cohort study. Crit Care Med (2021) 49(2):215–27. doi: 10.1097/CCM.0000000000004777 33372748

[5] Mostel Z Perl A Marck M Mehdi SF Lowell B Bathija S . Post-sepsis syndrome - an evolving entity that afflicts survivors of sepsis. Mol Med (2019) 26(1):6. doi: 10.1186/s10020-019-0132-z 31892321 PMC6938630

[6] Gotts JE Matthay MA . Sepsis: Pathophysiology and clinical management. BMJ (2016) 353:i1585. doi: 10.1136/bmj.i1585 27217054

[7] Singer M Deutschman CS Seymour CW Shankar-Hari M Annane D Bauer M . The third international consensus definitions for sepsis and septic shock (Sepsis-3). JAMA (2016) 315(8):801–10. doi: 10.1001/jama.2016.0287 PMC4968574 26903338

[8] Nedeva C Menassa J Puthalakath H . Sepsis: Inflammation is a necessary evil. Front Cell Dev Biol (2019) 7:108. doi: 10.3389/fcell.2019.00108 31281814 PMC6596337

[9] Parsons SA Greer PA . The Fps/Fes kinase regulates the inflammatory response to endotoxin through down-regulation of Tlr4, nf-κb activation, and tnf-α secretion in macrophages. J Leukocyte Biol (2006) 80(6):1522–8. doi: 10.1189/jlb.0506350 16959897

[10] Zirngibl RA Senis Y Greer PA . Enhanced endotoxin sensitivity in Fps/Fes-null mice with minimal defects in hematopoietic homeostasis. Mol Cell Biol (2002) 22(8):2472–86. doi: 10.1128/mcb.22.8.2472-2486.2002 PMC133716 11909942

[11] Xu S Liu X Bao Y Zhu X Han C Zhang P . Constitutive mhc class I molecules negatively regulate tlr-triggered inflammatory responses Via the fps-Shp-2 pathway. Nat Immunol (2012) 13(6):551–9. doi: 10.1038/ni.2283 22522491

[12] Clark SR Ma AC Tavener SA McDonald B Goodarzi Z Kelly MM . Platelet Tlr4 activates neutrophil extracellular traps to ensnare bacteria in septic blood. Nat Med (2007) 13(4):463–9. doi: 10.1038/nm1565 17384648

[13] Greer P . Closing in on the biological functions of Fps/Fes and fer. Nat Rev Mol Cell Biol (2002) 3(4):278–89. doi: 10.1038/nrm783 11994747

[14] Lei W Liu D Sun M Lu C Yang W Wang C . Targeting Stat3: A crucial modulator of sepsis. J Cell Physiol (2021) 236(11):7814–31. doi: 10.1002/jcp.30394 33885157

[15] Clere-Jehl R Mariotte A Meziani F Bahram S Georgel P Helms J . Jak-stat targeting offers novel therapeutic opportunities in sepsis. Trends Mol Med (2020) 26(11):987–1002. doi: 10.1016/j.molmed.2020.06.007 32631717

[16] Sangrar W Senis Y Samis JA Gao Y Richardson M Lee DH . Hemostatic and hematological abnormalities in gain-of-Function Fps/Fes transgenic mice are associated with the angiogenic phenotype. J Thromb Haemost (2004) 2(11):2009–19. doi: 10.1111/j.1538-7836.2004.00956.x 15550033

[17] Simmons J Pittet JF . The coagulopathy of acute sepsis. Curr Opin Anaesthesiol (2015) 28(2):227–36. doi: 10.1097/ACO.0000000000000163 PMC4407200 25590467

[18] Gattinoni L Brazzi L Pelosi P Latini R Tognoni G Pesenti A . A trial of goal-oriented hemodynamic therapy in critically ill patients. Svo2 collaborative group. N Engl J Med (1995) 333(16):1025–32. doi: 10.1056/NEJM199510193331601 7675044

[19] Senis YA Sangrar W Zirngibl RA Craig AW Lee DH Greer PA . Fps/Fes and fer non-receptor protein-tyrosine kinases regulate collagen- and adp-induced platelet aggregation. J Thromb Haemost (2003) 1(5):1062–70. doi: 10.1046/j.1538-7836.2003.t01-1-00124.x 12871378

[20] McDonald B Davis RP Kim SJ Tse M Esmon CT Kolaczkowska E . Platelets and neutrophil extracellular traps collaborate to promote intravascular coagulation during sepsis in mice. Blood (2017) 129(10):1357–67. doi: 10.1182/blood-2016-09-741298 PMC5345735 28073784

[21] Shinde-Jadhav S Mansure JJ Rayes RF Marcq G Ayoub M Skowronski R . Role of neutrophil extracellular traps in radiation resistance of invasive bladder cancer. Nat Commun (2021) 12(1):2776. doi: 10.1038/s41467-021-23086-z 33986291 PMC8119713

[22] Koutroulis I Batabyal R McNamara B Ledda M Hoptay C Freishtat RJ . Sepsis immunometabolism: From defining sepsis to understanding how energy production affects immune response. Crit Care Explor (2019) 1(11):e0061. doi: 10.1097/cce.0000000000000061 32166242 PMC7063962

[23] Fajgenbaum DC June CH . Cytokine storm. New Engl J Med (2020) 383(23):2255–73. doi: 10.1056/nejmra2026131 PMC7727315 33264547

[24] Boomer JS To K Chang KC Takasu O Osborne DF Walton AH . Immunosuppression in patients who die of sepsis and multiple organ failure. JAMA (2011) 306(23):2594. doi: 10.1001/jama.2011.1829 22187279 PMC3361243

[25] Karamanavi E McVey DG Laan SW Stanczyk PJ Morris GE Wang Y . The fes gene at the 15q26 coronary-Artery-Disease locus inhibits atherosclerosis. Circ Res (2022) 131(12):1004–17. doi: 10.1161/CIRCRESAHA.122.321146 PMC9770135 36321446

[26] Zhang X Goncalves R Mosser DM . The isolation and characterization of murine macrophages. Curr Protoc Immunol (2008) 14:14.1.1–14.1.14. doi: 10.1002/0471142735.im1401s83 PMC2834554 19016445

[27] Swamydas M Lionakis MS . Isolation, purification and labeling of mouse bone marrow neutrophils for functional studies and adoptive transfer experiments. J Vis Exp (2013) 77):e50586. doi: 10.3791/50586 PMC3732092 23892876

[28] Leng SX McElhaney JE Walston JD Xie D Fedarko NS Kuchel GA . Elisa And multiplex technologies for cytokine measurement in inflammation and aging research. Biol Sci Med Sci (2008) 63(8):879–84. doi: 10.1093/gerona/63.8.879 PMC2562869 18772478

[29] Bergmann CB Beckmann N Salyer CE Hanschen M Crisologo PA Caldwell CC . Potential targets to mitigate trauma- or sepsis-induced immune suppression. Front Immunol (2021) 12:622601. doi: 10.3389/fimmu.2021.622601 33717127 PMC7947256

[30] Masuda S Shimizu S Matsuo J Nishibata Y Kusunoki Y Hattanda F . Measurement of net formation *in vitro* and *in vivo* by flow cytometry. Cytometry A (2017) 91(8):822–9. doi: 10.1002/cyto.a.23169 PMC5601186 28715618

[31] Nielsen SR Strobech JE Horton ER Jackstadt R Laitala A Bravo MC . Suppression of tumor-associated neutrophils by lorlatinib attenuates pancreatic cancer growth and improves treatment with immune checkpoint blockade. Nat Commun (2021) 12(1):3414. doi: 10.1038/s41467-021-23731-7 34099731 PMC8184753

[32] Shaffer JM Smithgall TE . Promoter methylation blocks fes protein-tyrosine kinase gene expression in colorectal cancer. Genes Chromosomes Cancer (2009) 48(3):272–84. doi: 10.1002/gcc.20638 PMC2648816 19051325

[33] Stortz JA Raymond SL Mira JC Moldawer LL Mohr AM Efron PA . Murine models of sepsis and trauma: Can we bridge the gap? ILAR J (2017) 58(1):90–105. doi: 10.1093/ilar/ilx007 28444204 PMC5886315

[34] Starr ME Steele AM Saito M Hacker BJ Evers BM Saito H . A new cecal slurry preparation protocol with improved long-term reproducibility for animal models of sepsis. PloS One (2014) 9(12):e115705. doi: 10.1371/journal.pone.0115705 25531402 PMC4274114

[35] Shrum B Anantha RV Xu SX Donnelly M Haeryfar SM McCormick JK . A robust scoring system to evaluate sepsis severity in an animal model. BMC Res Notes (2014) 7(1):233. doi: 10.1186/1756-0500-7-233 24725742 PMC4022086

[36] Li J-L Li G Jing X-Z Li Y-F Ye Q-Y Jia H-H . Assessment of clinical sepsis-associated biomarkers in a septic mouse model. J Int Med Res (2018) 46(6):2410–22. doi: 10.1177/0300060518764717 PMC6023044 29644918

[37] Barichello T Sayana P Giridharan VV Arumanayagam AS Narendran B Della Giustina A . Long-term cognitive outcomes after sepsis: A translational systematic review. Mol Neurobiol (2019) 56(1):186–251. doi: 10.1007/s12035-018-1048-2 29687346

[38] Mostel Z Perl A Marck M Mehdi SF Lowell B Bathija S . Post-sepsis syndrome – an evolving entity that afflicts survivors of sepsis. Mol Med (2020) 26(1):1–14. doi: 10.1186/s10020-019-0132-z PMC6938630 31892321

[39] Illendula M Osuru HP Ferrarese B Atluri N Dulko E Zuo Z . Surgery, anesthesia and intensive care environment induce delirium-like behaviors and impairment of synaptic function-related gene expression in aged mice. Front Aging Neurosci (2020) 12:542421. doi: 10.3389/fnagi.2020.542421 33088271 PMC7544741

[40] Muscedere J Maslove D Boyd JG O’Callaghan N Lamontagne F Reynolds S . Prevention of nosocomial infections in critically ill patients with lactoferrin (Prevail study): Study protocol for a randomized controlled trial. Trials (2016) 17(1):474. doi: 10.1186/s13063-016-1590-z 27681799 PMC5041570

[41] [Dataset] . Available at: https://www.ncbi.nlm.nih.gov/geo/geo2r/?acc=GSE118657.

[42] Rautanen A Mills TC Gordon AC Hutton P Steffens M Nuamah R . Genome-wide association study of survival from sepsis due to pneumonia: An observational cohort study. Lancet Respir Med (2015) 3(1):53–60. doi: 10.1016/s2213-2600(14)70290-5 25533491 PMC4314768

[43] Rogne T Damås JK Flatby HM Åsvold BO DeWan AT Solligård E . The role of fer Rs4957796 in the risk of developing and dying from a bloodstream infection: A 23-year follow-up of the population-based nord-trøndelag health study. Clin Infect Dis (2021) 73(2):e297–303. doi: 10.1093/cid/ciaa786 PMC8282309 32699877

[44] Udell CM Samayawardhena LA Kawakami Y Kawakami T Craig AWB . Fer and Fps/Fes participate in a Lyn-dependent pathway from fcϵri to platelet-endothelial cell adhesion molecule 1 to limit mast cell activation. J Biol Chem (2006) 281(30):20949–57. doi: 10.1074/jbc.m604252200 16731527

[45] Marshall JC . Why have clinical trials in sepsis failed? Trends Mol Med (2014) 20(4):195–203. doi: 10.1016/j.molmed.2014.01.007 24581450

[46] Maslove DM Tang B Shankar-Hari M Lawler PR Angus DC Baillie JK . Redefining critical illness. Nat Med (2022) 28(6):1141–8. doi: 10.1038/s41591-022-01843-x 35715504

[47] Su Y-X Xu L Gao X-J Wang Z-Y Lu X Yin C-F . Long-term quality of life after sepsis and predictors of quality of life in survivors with sepsis. Chin J Traumatol (2018) 21(4):216–23. doi: 10.1016/j.cjtee.2018.05.001 PMC6085193 30017545

